# Influence of oxygen on lovastatin biosynthesis by *Aspergillus terreus* ATCC 20542 quantitatively studied on the level of individual pellets

**DOI:** 10.1007/s00449-015-1366-y

**Published:** 2015-01-28

**Authors:** Marcin Bizukojc, Joanna Gonciarz

**Affiliations:** Faculty of Process and Environmental Engineering, Department of Bioprocess Engineering, Lodz University of Technology, ul. Wolczanska 213, 90-924 Lodz, Poland

**Keywords:** Mass transfer, Fungal pellets, Lovastatin, Oxygen microprobe, *Aspergillus terreus*

## Abstract

Despite oxygen is believed to be the most important environmental factor for any aerobic microbial process, the quantitative studies of its influence on growth and metabolite formation on the level of individual pellets formed by filamentous fungi were seldom performed. Never was it made for lovastatin producer *Aspergillus terreus* ATCC20542. Thus, this work is a quantitative study of oxygen transfer into *A. terreus* pellets during lovastatin biosynthesis in the shake flask culture. The basic measurement tool was an oxygen microprobe allowing for obtaining oxygen concentration profiles in the pellets. The pellets of various sizes from 1,600 to 6,400 μm exerting different oxygen transfer conditions were studied. Also various initial concentrations of carbon source were applied to change the conditions of biological reaction running in the pellets. Effective diffusivities in *A. terreus* pellets ranged from 643 to 1,342 μm s^−1^ dependent on their size and structure. It occurred that only the smallest pellets of diameter equal to about 1,400 μm were fully penetrated by oxygen. What is more, apart from the size of pellets, the appropriate lactose concentration was required to effectively produce lovastatin. Its value was correlated with oxygen concentration on the surface of the pellet and could not be either too high, as the aforementioned oxygen level tended then to zero, or too low, as despite high oxygen concentration no biological reaction ran in the pellet and no lovastatin was formed.

## Introduction

Lovastatin is a polyketide secondary metabolite from *Aspergillus terreus*. It plays an important role in medicine being a competitive inhibitor of S-3-hydroxymethylglutaryl-CoA reductase and decreasing in this way the level of endogenous cholesterol in human organisms.

As lovastatin is produced by filamentous fungi, i.e. strictly aerobic organisms, its formation is affected by oxygen saturation in the cultivation broth [1]. Furthermore, the formation of pellets of various sizes by *A. terreus* in the submerged culture and the fact that fungal suspensions may have changeable rheological properties during the cultivation make oxygen transfer processes one of the most important issues to be studied for the efficient lovastatin formation. In several papers, mass transfer coefficients between gas and liquid phase were determined [2–5]. Also, the effect of aeration or fungal morphology on lovastatin formation was sometimes discussed with regard to diffusion in the pellets [1, 6]. Upon the experimental data and general knowledge, many authors attributed better lovastatin formation to smaller pellets and better oxygen transport into pellets [1, 6, 7] although Casas Lopez et al. [2] reached contradictory conclusions. The importance of oxygen for lovastatin formation made several researchers aerate their pelleted cultures even with oxygen-enriched air [2, 3, 8]. Nevertheless, Bizukojc and Ledakowicz [9] and Lai et al. [1, 10] claimed that too much oxygen was not favourable for lovastatin formation.

What is more, hardly ever was the effect of oxygen transfer, especially diffusion in the pellets, in conjunction with *A. terreus* morphology, i.e. pellet size, studied in the quantitative way on the level of the individual pellets during lovastatin biosynthesis. Only some limited data were supplied by Gonciarz and Bizukojc [11]. Furthermore, upon general biochemical engineering knowledge, it is known that in the microbial systems not only oxygen transfer but also the supply of carbon substrate and the correlation between the amount of carbon substrate and oxygen are of high importance. It also deals with lovastatin, whose formation is to the highest extent carbon source dependent [12–14]. Unfortunately, those authors, who most intensively studied the effect of oxygen on lovastatin biosynthesis, did not supply any data concerning the utilisation of the carbon substrate used [2, 3, 8, 15]. Therefore, never was the issue of carbon substrate and oxygen utilisation by *A. terreus* simultaneously studied and that is why it is impossible to find the unequivocal rule concerning the effect of oxygen on lovastatin biosynthesis.

Although not studied in *A. terreus*, the issue of diffusion of oxygen in fungal pellets with regard to other fungal species was previously the object of research. Oxygen concentration measurements with the use of microprobe in the pellets of *Penicillium chrysogenum* and modelling of mass transfer were, for example, performed by Wittler et al. [16]. Oxygen profiles in the pellets of an α-amylase producer *A. niger* were measured by Hille et al. [17]. Several works concerning modelling pelleted growth of filamentous fungi, in which oxygen utilisation and transfer (diffusion) terms were included, can be found. Cui et al. [18] modelled *A. awamori* growth taking diffusion, shaving intensity (changes of fungal morphology) and autolysis rate of biomass into account. Hellendoorn et al. [19] measured oxygen concentration profiles in natural and artificial (made of agar) pellets of *A. awamori* and performed the advanced mathematical modelling of their growth including the determination of Thiele number. Also, Michel et al. [20] made measurements of oxygen level together with the mathematical description of oxygen transfer in the fungal pellets but in this case they worked with a lignin degrading basidiomycete *Phanerochaete chrysosporium*. Recently, the methods (morphological engineering techniques) to facilitate mass transfer and maximise metabolite formation (enzymes) in the fungal cultures were also proposed [21].

To sum up, all aforementioned works indicate on the importance of oxygen transfer for growth and metabolite formation in filamentous fungi, when pellets are formed. So there is a need to find the quantitative description of oxygen transfer and uptake processes and carbon substrate utilisation in pellets of filamentous fungi. In the case of lovastatin producer *A. terreus*, it is going to be made here for the first time.

Thereby, the aim of this work was to quantify oxygen transfer processes in *A. terreus* pellets during their growth and estimate its effect on lovastatin production. Furthermore, the utilisation of carbon substrate in association with oxygen supply will be taken into account. It is going to be made on the level of the individual pellets, which is a different approach than that met in literature concerning lovastatin biosynthesis by *A. terreus*.

## Materials and methods

### Strain, media and culture conditions

*Aspergillus terreus* ATCC 20542 was employed in the experiments. It was cultivated in shake flasks at 30 °C and at the constant rotation speed of 110 min^−1^. The total and working volumes of flasks were 500 and 157 ml, respectively [6]. The preculture was prepared from 10-day malt extract slants. The spores were washed with the sterile medium to obtain various numbers of spores in the preculture and, as a consequence, pellets of various diameters in the production culture [6].

The compositions of the preculture and production media were the same as used by Gonciarz and Bizukojc [11] with lactose as the sole carbon source and yeast extract as the sole nitrogen source. Eight experiments were parallel performed with various mean pellet diameter from 1,400 to 6,200 μm and initial lactose concentrations from 2.5 to 40 g l^−1^. Also, the additional independent experiment to obtain oxygen profiles in the viable and deactivated pellets was made.

### Analysis of cultivation broths

Lovastatin (LOV) and lactose (LAC) concentrations were determined by liquid chromatography (UPLC^®^, Waters, USA). Organic nitrogen (N) was determined with the use of carbon and nitrogen analyser IL550TOC-TN (HACH, USA) and biomass (X) was assayed as dry weight [11]. The accuracy of the applied analytical techniques was as follows. For biomass, the error ranged from 5 to 15 %, for lactose, it did not exceed 4 %, for lovastatin, it was lower than 1 % and for organic nitrogen, it was around 3 %.

### Measurement of oxygen profiles in the pellets

Several dozen fresh pellets were poured onto an 8-cm Petri dish with the blue-stained agar and flooded with the filtered cultivation broth. The colour of the agar facilitated the observation of the yellowish pellets and agar prevented the glass oxygen sensor from breaking. The measurements were performed with the use of the 10 μm tip glass oxygen sensor (Clark-type probe) mounted on the computer controlled (SensorTrace PRO v3.0.2) motorised micromanipulator (UNISENSE, Denmark). Detection limit of the microprobe was 0.3 μM (0.01 mg O_2_ l^−1^) and the accuracy of the measurements was estimated upon the fivefold measurement of the same experimental point to be equal to ±0.03 mg O_2_ l^−1^. The sensor was calibrated in the air-saturated water and the solution containing 2 g of sodium ascorbate in 0.1 M NaOH (oxygen-free solution).

As the broth containing viable pellets was immediately (within less than 1 min) taken out of the shake flask and owing to the high area of contact of the filtered broth with air, oxygen concentration in the broth was close to the maximum value. Any oxygen deficiency that may have negatively influenced on the measurements was then avoided. It was checked by measuring oxygen concentration at various depths in the “clear” broth, i.e. in the distance of several centimetres from the pellets. Oxygen concentration profiles were quickly measured in three randomly selected pellets from each sample. The time of the individual measurement for each pellet was determined by the technical capabilities of the used microprobe. Each experimental point was obtained within 4 s. The microprobe waited in the programmed position for 2 s (stabilisation time) and the readout lasted next 2 s. The moving time of the microprobe between two neighbouring measurement points was negligible (less than 0.3 s).

To get oxygen profiles for the dead (deactivated) pellets, they were treated with glutaraldehyde solution before oxygen concentration measurements. The measuring procedure was exactly the same, especially its time, as in the case of the viable pellets to have comparable results.

### Image analysis techniques

All pellets subjected to oxygen profile measurements were digitally photographed. In each image, a scale bar of the known length was also included. Pellet diameter was then manually measured with the use of image analysis software MicroImage 4.0 (Media Cybernetics, USA). This technique allowed for the discrimination of oxygen concentration points measured in the boundary layer and inside the pellets.

### Calculations

Volumetric rates were calculated as follows. First, the experimental data (concentration vs. time) were approximated with the spline third-order polynomial functions. The smoothed data were then subjected to the numerical differentiation to obtain the changes of a given rate in time. PTC Mathcad 14 and Originlab Origin software were used for this purpose. The second-order differential equation describing the diffusion of oxygen into pellets was solved (Adams–Bashford method) and its parameters identified (non-linear Levenberg–Marquardt method) with the use of PTC Mathcad 14 software.

## Results and discussion

### Lovastatin biosynthesis vs. oxygen transfer to *A. terreus* pellets

**Fig. 1:**
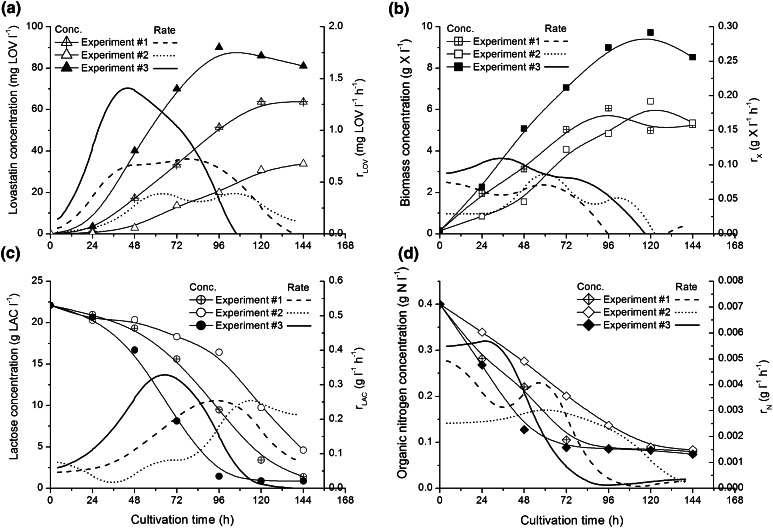
Changes of lovastatin (**a**), biomass (**b**), lactose (**c**) and organic nitrogen (**d**) concentrations in time in experiments from #1 to #3 with varied pellet diameter; linear graphs present lovastatin *r*_LOV_ and biomass *r*_X_ volumetric formation rates and lactose *r*_LAC_ and organic nitrogen *r*_N_ volumetric uptake rates

In the first three experiments, due to the use of various numbers of spores in the preculture, the pellets of the following mean diameters (and standard deviations) were obtained. In experiment #1 it was 3,700 (±400) μm, in experiment #2: 6,200 (±900) μm and in experiment #3: 1,400 (±200) μm. In Fig. 1, volumetric rates of lactose uptake, lovastatin formation and biomass growth for these experiments are shown, so are the concentrations of these broth components.

It is clearly seen in Fig. 1a that the smallest pellets obtained in experiment #3 occurred to be the most favourable for biosynthesis of lovastatin, whose both titre (above 80 mg LOV l^−1^) and maximum volumetric formation rate (almost 1.5 mg LOV l^−1^ h^−1^) were the highest. This rate was twice higher than that in the run with medium-sized pellets and more than three times higher than that in experiment #2, in which the largest pellets grew. The best lovastatin titres for the smallest pellets would not be obtained, if it was not for the highest biomass amount and volumetric biomass growth rate, almost 10 g X l^−1^ in the idiophase (biomass growth stationary phase) and around 0.1 g l^−1^ h^−1^ in the trophophase (biomass growth phase), respectively (Fig. 1b). In the systems with smaller pellets more biomass is formed, which is favourable for mixed growth associated lovastatin formation [6]. Also, fast lactose utilisation contributed to high lovastatin titre as lovastatin formation is strongly dependent on the available carbon source [12, 14]. In experiment #3, lactose concentration approached zero already in 96 h, while in the experiment #1 it happened about 144 h and in experiment #2 with the largest pellets lactose was not ultimately utilised. Also, the maximum values of volumetric lactose uptake rate confirmed the aforementioned observations. The highest out of all measured values (0.3 g LAC l^−1^ h^−1^) was observed already before 72 h in experiment #3, while in the other runs these maxima were delayed and had lower values (Fig. 1c). Also, the effect of *A. terreus* pellet size was seen with regard to organic nitrogen uptake kinetics (Fig. 1d). As it was for lactose, organic nitrogen concentration decreased the fastest in the system with the smallest pellets.

All these findings described above confirmed the behaviour of *A. terreus* culture with pellets of various sizes described previously by Bizukojc and Ledakowicz [6]. Biomass growth curve in *A. terreus* requires, however, more attention. Pawlak and Bizukojc [22] reported that the trophophase in *A. terreus* consisted of exponential growth phase and linear growth phase. Exponential growth phase was very short and lasted not more than 12 h and it was the time, when pellets were extremely small. Their diameter was lower than 500 μm [19]. In the present experiments, although exponential growth phase must have occurred, only distinct linear biomass growth is seen in Fig. 1b. Here, the sampling was not made more frequently in the first 24 h. Nevertheless, the two-phase trophophase in *A. terreus* lovastatin biosynthetic system needs to be more thoroughly analysed on the pellet level. Here, the intuition supported by general chemical engineering knowledge could prompt that in the smallest pellets all substrates, including oxygen, were easier and faster transferred into pellets [6]. But the linear growth phase was the evidence that mass transfer limitation in the culture occurred [23]. Minimising this limitation might lead to the further improvement of lovastatin formation and it is required to find the quantitative description of transfer of substrates, above all oxygen, into *A. terreus* pellets.

**Fig. 2:**
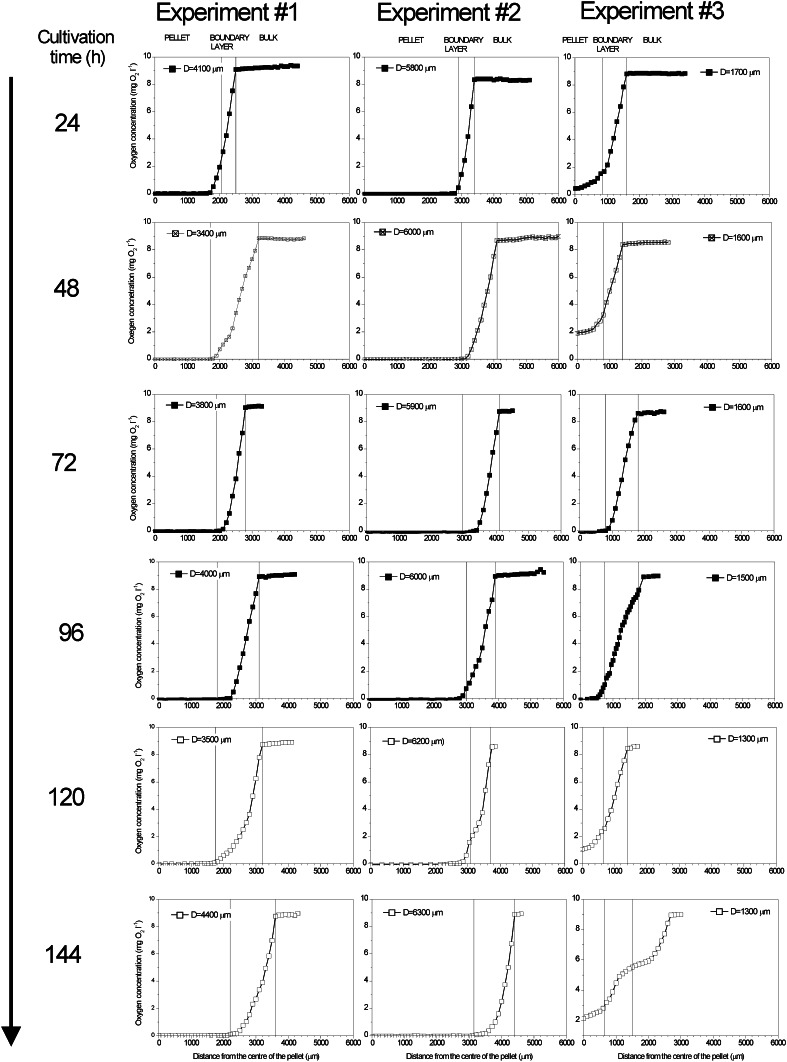
Averaged experimental oxygen profiles for *A. terreus* viable pellets of various diameters; oxygen concentrations in the pellet, boundary layer and bulk liquid were discriminated

Therefore, it is going to be quantitatively proved here that it was for oxygen transfer into *A. terreus* pellets that smaller pellets had more intensive metabolism and produced more lovastatin. Oxygen, not carbon and nitrogen substrates, was more important here due to several reasons. First of all, oxygen is the most difficult substrate (or rather one should say final electron acceptor) to be supplied to any aerobic cultures. Its solubility in water and cultivation media is limited (about 8 mg O_2_ l^−1^) and it must be continuously supplied by shaking or by aeration devices. That is why oxygen limitation most likely occurred in the studied fungal system. Second, even if oxygen were effectively supplied to the broth owing to high convective mass transfer coefficients, *k*_*L*_ *a*, obtained in the well-mixed and aerated flasks or bioreactors, it would have to somewhat reach the fungal cells. In the case of pelleted morphology of *A. terreus*, there are potentially two additional limiting steps, i.e. mass transfer resistance in the film surrounding the pellet (described by convective mass transfer coefficient *k*_S_ *a*) and intrapellet diffusion steps (described by effective diffusivity *D*_eff_ as the fungal pellets are impermeable to flow). The quantification of these limiting steps was possible using microprobe technique to measure oxygen concentration in the film surrounding the pellets and in the pellets themselves. For this purpose, in this study, oxygen concentration profiles were measured in the viable and deactivated pellets. The averaged oxygen profiles in the viable pellets for the three performed experiments are shown in Fig. 2.

It is clearly seen that the shapes of oxygen profiles depended on the time of sampling and pellet diameter in the given experiment. In 24 h of the experiments, *A. terreus* was already in the linear growth phase, i.e. mass transfer (oxygen) limitation took place. Both in experiment #1 and #2, in this early moment of the cultivation, oxygen concentration on the level close to 0 mg O_2_ l^−1^ was observed inside the pellets. In experiment #1, oxygen was found in the pellets not deeper than at 300 μm. What is more, in the largest pellets from experiment #2, oxygen concentration approached zero already in the boundary layer. Oxygen was usually detected in these pellets not deeper than at 100 μm. These oxygen levels in the pellets indicated on the microaerobic, unfavourable for lovastatin formation, conditions inside the pellets as the driving force for diffusion drastically decreased, practically down to zero. What is more, 24 h later in experiment #2, oxygen concentration distinctly dropped down to zero in the boundary layer. In the smallest pellets from experiment #3, oxygen concentration was close to zero only in the centre of the pellet and almost the whole pellet was penetrated by oxygen. This state was actually observed to the end of the experiment. From 48 h, in experiment #2, no oxygen concentration was found inside the pellets and it remained like that practically to the end of experiment. The similar situation took place in the run with the medium-size pellets, although in this case, low oxygen concentrations were found in the external layers of the pellets. In the later hours of the run, the profiles became less accurate due to the lack of nutrients, causing changes in the structure of the pellet, which was seen in 144 h of experiment #3 (Fig. 2).

**Fig. 3:**
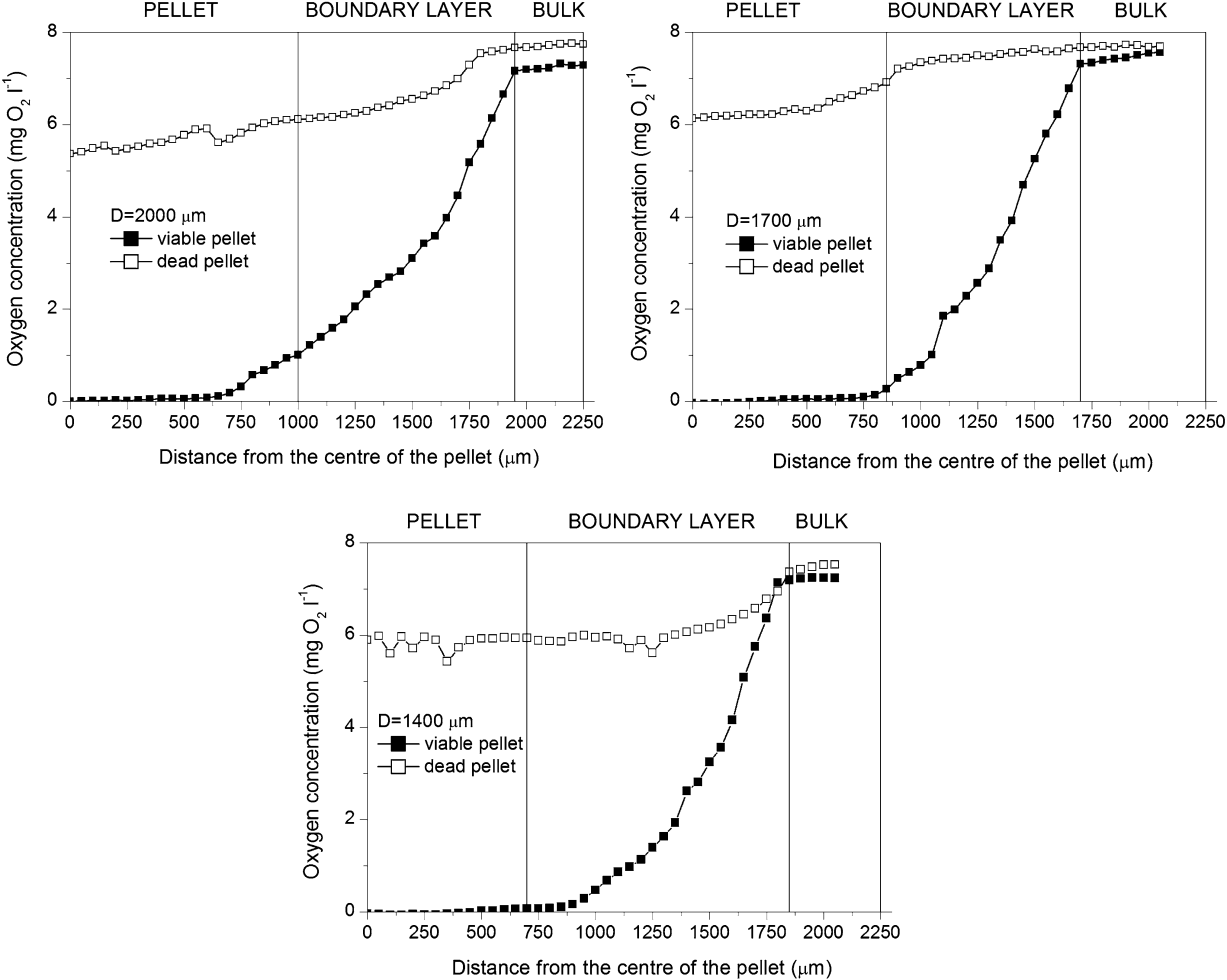
Comparison of oxygen profiles in viable and dead pellets of various diameters in 96 h of the experiment; the measuring conditions for both viable and dead cells with regard to measurement time were exactly the same

Additionally, it was studied, what would happen when there was no oxygen uptake in the pellets. For this purpose, oxygen profiles were measured in the viable and deactivated (dead) pellets of diameters 2,000, 1,700 and 1,400 μm, respectively, obtained in the independent experiment (Fig. 3). It is clearly seen that even in the deactivated pellets, oxygen concentration decreased towards pellet centre. There was a decrease in the boundary layer and in the pellets themselves. In the largest tested pellets (2,000 and 1,700 μm), oxygen concentration inside the pellets was lower than that in the boundary layer, while for the smallest tested pellets (1,400 μm) its value was almost the same as in the boundary layer. As the time of measurements was exactly the same as for the viable pellets, the full penetration of the deactivated pellets by oxygen as could be expected in the steady state was not observed. The resistance of diffusion, here not influenced by oxygen uptake, was clearly seen.

Due to the fact that the measurement of oxygen profiles took place in the flow quiescence conditions, i.e. there was no flow of the medium around the pellets (as described in “Materials and methods”), oxygen transfer in the boundary layer (seen both in Figs. 2, 3) had to be taken into account too, which will be described in next sections. All in all, it was ultimately confirmed that oxygen transfer limitations influenced the overall run of the cultivation seen in Fig. 1.

### Quantitative description of internal mass transfer limitation in *A. terreus* pellets

A shell model for a biocatalyst (a fungal pellet here) was used to find the effective diffusivity for oxygen in *A. terreus* pellets. The assumptions of this model were as follows:

- Pellets were isothermal as temperature gradients generated by fungal cells were negligible and the performed microprobe measurements were fast,
- Mass transfer occurred by diffusion only as pellets were impermeable to flow,
- Diffusion was described by Fick’s law with the constant effective diffusivity; interaction of oxygen with other concentration gradients and phenomena affecting transport of charged species were ignored; effective diffusivity was constant and independent of oxygen concentration in the pellet either,
- Although difficult to be fulfilled for fungal pellets due to cell differentiation, pellets were treated as homogeneous,
- Oxygen partition coefficient was unity and ensured that there was no discontinuity of concentration at the solid–liquid interface,
- Pellets were at the steady state; it was valid, if there was no change in the activity of the pellet, e.g. due to enzyme deactivation, cell growth or differentiation; due to slow growth and differentiation of fungal cells in the short time intervals steady state could be assumed.
- Oxygen concentration varied only with a single spatial variable and diffused radially through the pellet from the external surface towards its centre.

The solution of oxygen balance for the individual pellet in accordance with the aforementioned assumptions resulted in Eq. 1:

1 $$D_{\text{eff}} \left( {\frac{{{\text{d}}^{2} c_{{{\text{O}}_{ 2} }} }}{{{\text{d}}r^{2} }} \cdot r^{2} + 2 \cdot r \cdot \frac{{{\text{d}}c_{{{\text{O}}_{ 2} }} }}{{{\text{d}}r}}} \right) - r_{{{\text{O}}_{ 2} }} \cdot r^{2} = 0$$

where: *D*_eff_ effective diffusivity (μm^2^ s^−1^), *r* pellet radius (μm), $c_{{{\text{O}}_{ 2} }}$ oxygen concentration (mg O_2_ l^−1^) and $r_{{{\text{O}}_{ 2} }}$ biological reaction rate (mg O_2_ l^−1^ s^−1^).

The integration of this equation allowed for obtaining the changes of oxygen concentration versus pellet radius, provided the equation for biological reaction rate was used. In the case of the immobilised biocatalysts such as fungal pellets, biological kinetics is the most often expressed by one out of three relationships. Biological reaction can be either of zero-order (independent of substrate concentration), or of first-order (directly proportional in the first power to substrate concentration) or be expressed by Michaelis–Menten equation. In this work, the latter kinetics was used, so:

2 $$r_{{{\text{O}}_{ 2} }} = r_{\hbox{max} } \frac{{c_{{{\text{O}}_{ 2} }} }}{{c_{{{\text{O}}_{ 2} }} + K_{\text{M}} }}$$

where: *r*_max_ maximum reaction rate (mg O_2_ l^−1^ s^−1^) and *K*_M_ Michaelis constant (mg O_2_ l^−1^).

Biological kinetics parameters were previously determined by Gonciarz and Bizukojc for *A. terreus* pellets growing in the same conditions: lactose as the carbon source and the same shaking speed [11]. They equalled *r*_max_ = 0.026 mg O_2_ l^−1^ s^−1^ and *K*_M_ = 0.11 mg O_2_ l^−1^ and were also used in this study.

The experimental data from Fig. 2 were used to find the effective diffusivities in pellets of various diameters. They are all collected in Table 1.

**Table 1:** Averaged effective diffusivities, external efficiencies and their standard deviations for *A. terreus* pellets of various sizes from various hours of cultivation

Experiment/mean pellet diameter *D*	Hour of cultivation (h)	Effective diffusivity *D*_*eff*_ (μm^2^ s^−1^)^a^	External efficiency *η*_*e,m*_
#1 *D* = 3,700 (±400) μm	24	634 ± 94	0.93 ± 0.05
	48	1,164 ± 78	0.035 ± 0.005
	72	896 ± 47	0.0009 ± 0.0005
	96	887 ± 29	0.0009 ± 0.0004
	120	1155 ± 151	0.76 ± 0.18
	144	1342 ± 120	0.56 ± 0.34
#2 *D* = 6,200 (± 900) μm	24	993 ± 28	0.26 ± 0.05
	48	789 ± 45	0.29 ± 0.06
	72	850 ± 48	0.0009 ± 0.0005
	96	1,027 ± 150	0.60 ± 0.06
	120	824 ± 115	0.65 ± 0.06
	144	925 ± 142	0.33 ± 0.05
#3 1,400(±200) μm	24	985 ± 11	0.93 ± 0.03
	48	985 ± 20	0.97 ± 0.01
	72	909 ± 66	0.60 ± 0.05
	96	918 ± 120	0.92 ± 0.03
	120	1,691 ± 210	0.97 ± 0.04
	144	1,061 ± 123	0.98 ± 0.06

^a^the values of effective diffusivities were less accurately determined for these pellets, in which low oxygen concentration was observed

Generally, the differences in the values of effective diffusivities were not too high. It is known from literature that this parameter is strictly connected with the structure of the fungal pellet, its density and porosity (both varied with the time of cultivation and dependent on cultivation conditions), and to a certain extent its diameter [11, 17, 24]. It was the reason that there were no simple correlations to be found from Table 1. Bizukojc and Ledakowicz [6] previously found that smaller pellets (theoretically they should have higher diffusivities) were usually denser (it may at the same time decrease diffusivity) than the larger ones. The smaller pellets were denser due to better substrate availability and from the cultures with small pellets higher biomass harvest was obtained, which is seen in Fig. 1b and was reported by Bizukojc and Ledakowicz [6]. Nevertheless, it was observed that in the later stage of the cultivation the diffusivity generally increased (Table 1) as pellet structure got looser, which was connected with the degradation of hyphae due to lactose deficiency and ageing of mycelium (Fig. 1c). This phenomenon was previously visualised by Bizukojc and Ledakowicz [6]. Also, in experiment #3, the values of diffusivities were generally higher as smaller pellets were then obtained (Table 1).

Both size (pellet diameter) and structure (biomass profile) of the pellets influenced on the values of the effective diffusivities and it is difficult to estimate their contribution. So the values of diffusivities alone occurred insufficient to conclude about the rate of the processes run in *A. terreus* pellets and its limiting steps. Furthermore, in practice, it is more important to know, in which growth conditions mass transfer resistance may influence on the overall run of the process with *A. terreus*. Thus, the effective diffusivities had to be associated with biological reaction kinetics to find whether the processes in the pellets ran in the kinetic or diffusive regime. For this purpose, the correlation between observable Thiele modulus (Weisz’s modulus) and internal efficiency was calculated upon the experimental data. This correlation took oxygen concentration on the surface of the pellet into account (see Eq. 3 and “Appendix”) and owing to oxygen microprobe measurement and image analysis this value was well known in all described experiments.

Weisz’s modulus *Φ* was calculated from Eq. 3:

3 $$\varPhi = 2 \cdot \phi_{\text{m}}^{2} \cdot \eta_{{{\text{i}},{\text{m}}}} \cdot \left( {1 + \beta } \right) \cdot \left[ {1 + \beta \ln \left( {\frac{\beta }{1 + \beta }} \right)} \right]$$

and internal efficiency *η*_i,m_ from Eq. 4 [25]:

4 $$\eta_{\text{i,m}} = \frac{{\eta_{{{\text{i,}}0}} + \beta \cdot \eta_{{{\text{i,}}1}} }}{1 + \beta }$$

**Fig. 4:**
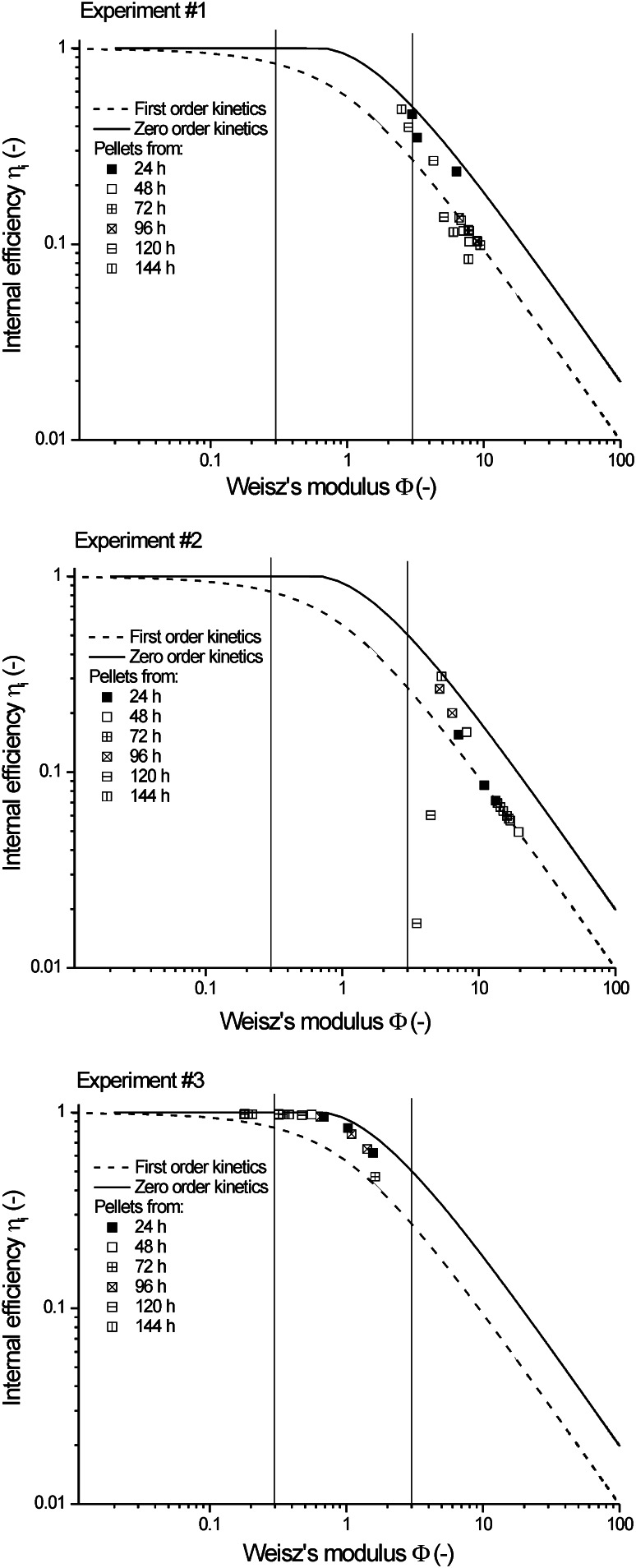
Determination of mass transfer impact on the processes run in *A. terreus* pellets using Weisz’s modulus and criterion; data for experiments from #1 to #3; vertical lines denounce Weisz’s criterion *Φ* < 0.3, 0.3 < *Φ* < 3 and *Φ* > 3, respectively

The symbols in Eqs. 3 and 4 were defined in the Appendix for the sake of brevity. The results of these calculations are depicted in Fig. 4. All points were calculated for Michaelis–Menten kinetics. Additionally, the simulated curves for zero- and first-order kinetics were included in this figure as linear graphs. It is clearly seen that even for the smallest pellets studied (experiment #3), it cannot be unequivocally said that mass transfer limitation with regard to oxygen was negligible, as the majority of points fitted into the region 0.3 < *Φ* < 3. In accordance with Weisz’s criterion only at *Φ* < 3, oxygen transfer limitation would be insignificant. For the other two experiments, oxygen transfer limitation was significant as *Φ* exceeded three.

It is clearly seen from these results that it is not easy to assure the conditions, in which lovastatin biosynthesis by *A. terreus* would run in purely kinetic regime. Pellet diameter would probably have to be as low as the ones during exponential growth phase studied by Pawlak and Bizukojc [22]. In practice, it is difficult to hold pellets small due to usual biomass growth. The run of lovastatin biosynthesis, under the conditions when *Φ* > 3 and mass transfer limitation are significant, is completely ineffective.

### Quantitative description of external mass transfer limitation in *A. terreus* pellets

As the internal efficiency of the biological reaction and diffusion in *A. terreus* pellets was dependent on oxygen concentration on the surface of the pellet (see Eq. 3 and “Appendix”), the full quantitative analysis of oxygen transfer was supplemented with the estimation of external mass transfer limitation. Thereby, the values of convective mass transfer coefficient *k*_*S*_ *a* were found. It must be, however, remembered that under the measurement conditions (quiescent, without any flow), convective mass transfer was the least efficient and the determined values of *k*_*S*_ *a* were the lowest possible for the studied system.

The calculation of *k*_*S*_ *a* was made upon the dimensionless Sherwood number.

5 $${\text{Sh}} = \frac{{k_{\text{S}} \cdot D}}{{D_{\text{eff}} }}$$

Under flow quiescent conditions, Sherwood number is equal to 2 [26]. As convective mass transfer took place in the liquid film, effective diffusivity of the medium equal to 2,800 μm^2^ s^−1^ was assumed [24]. Calculation of the specific mass transfer area *a* was made upon the known pellet diameter *D*:

6 $$a = \frac{{A_{\text{pellet}} }}{{V_{\text{pellet}} }} = \frac{6}{D}$$

**Fig. 5:**
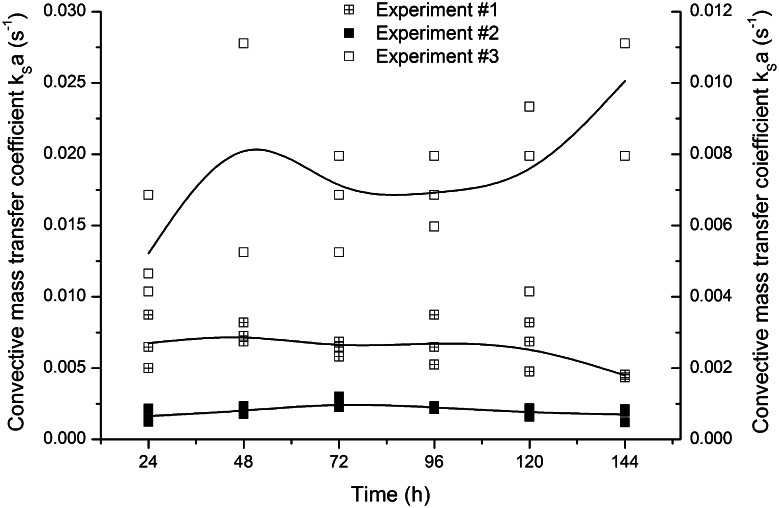
Changes of *k*_*S*_ *a* in time for *A. terreus* pellets in the experiments from #1 to #3; values were determined in each hour of the cultivation for three measured pellets

In Fig. 5, the changes of convective oxygen transfer coefficient *k*_S_ *a* are shown in the function of time of cultivation. As expected, the highest values were obtained for experiment #3, the one with the smallest pellets, as convective mass transfer coefficient *k*_*S*_ is inversely proportional to particle diameter *D* (upon transformed Eq. 4) and mass transfer area *a* (Eq. 5).

Additionally, the external efficiencies, dependent on pellet diameter and hour of the cultivation, were calculated from Eq. 7:

7 $$\eta_{\text{e,m}} = \frac{{c_{{{\text{O}}_{ 2} }}^{\text{S}} \cdot \left( {K_{\text{M}} + c_{{{\text{O}}_{ 2} }}^{\text{B}} } \right)}}{{c_{{{\text{O}}_{ 2} }}^{\text{B}} \cdot \left( {K_{\text{M}} + c_{{{\text{O}}_{ 2} }}^{\text{S}} } \right)}}$$

where: $c_{{{\text{O}}_{ 2} }}^{\text{B}}$ bulk oxygen concentration (mg O_2_ l^−1^) and $c_{{{\text{O}}_{ 2} }}^{\text{S}}$ oxygen concentration on the surface of the pellet (mg O_2_ l^−1^) The values of external efficiencies are also presented in Table 1. As above, Michaelis–Menten kinetics was used to express biological reaction rate.

Taking into consideration both internal and external efficiencies, few important observations regarding the influence of oxygen on lovastatin formation were made.

In experiment #3 (pellet diameter 1,400 μm), in which diffusive oxygen transfer limitation in the pellets was the lowest (0.3 < *Φ* < 3), the highest lovastatin titres and formation rates were achieved. It also coincided with mainly over 90 % external efficiency of oxygen transfer. So it was for the sufficient oxygen concentration on the surface of the pellets and inside them that lovastatin could be efficiently formed.

Analysing all these data concerning oxygen transfer obtained in this study, it must be remembered that due to the technical limitations of oxygen concentration measurements (flow quiescent conditions) they do not exactly reflect any stirred system, either shake flask or bioreactor. But upon general chemical engineering rules, one knows that the conditions of oxygen transfer in the stirred systems are always better than the quiescent ones. Above all, the boundary layer is thinner. It facilitates oxygen convection and increases the driving force for diffusion, which is in the real stirred systems higher than that measured in this study. Here, the least efficient mass transfer conditions were actually studied. But too optimistic conclusions could not be drawn upon it.

Pellet diameter determined the conditions of oxygen transfer and the availability of oxygen determined lactose utilisation. It is worth noticing that more or less in the end of trophophase (between 48 and 96 h, dependent of the run) oxygen transfer must have most intensively influenced the run of the process as both determined effective diffusivities and external efficiencies lowered (Table 1). It was the moment, in which lovastatin production rate was the highest and so was lactose utilisation rate (Fig. 1a, c). At that time high oxygen supply was undoubtedly required, as it is for lactose (carbon substrate) utilisation that lovastatin is fast and effectively produced [9, 12, 14]. It is connected with the biochemical mechanism ruling lovastatin formation as lovastatin polyketide synthases (PKS) require high fluxes of acetyl-CoA and malonyl-CoA (formed by carboxylation of acetyl-CoA). Furthermore, the PKS intermediate, i.e. 4a,5-dihydromonacolin, is later oxidised with molecular oxygen to mevinolinic acid (lovastatin).

Therefore, apart from pellet diameter, another factor will be studied here in the context of oxygen transfer in the pellets. It is carbon substrate (lactose), whose concentration changes to the high extent and may easily become limiting in the batch system.

### Effect of lactose concentration on oxygen concentration in the pellets

**Fig. 6:**
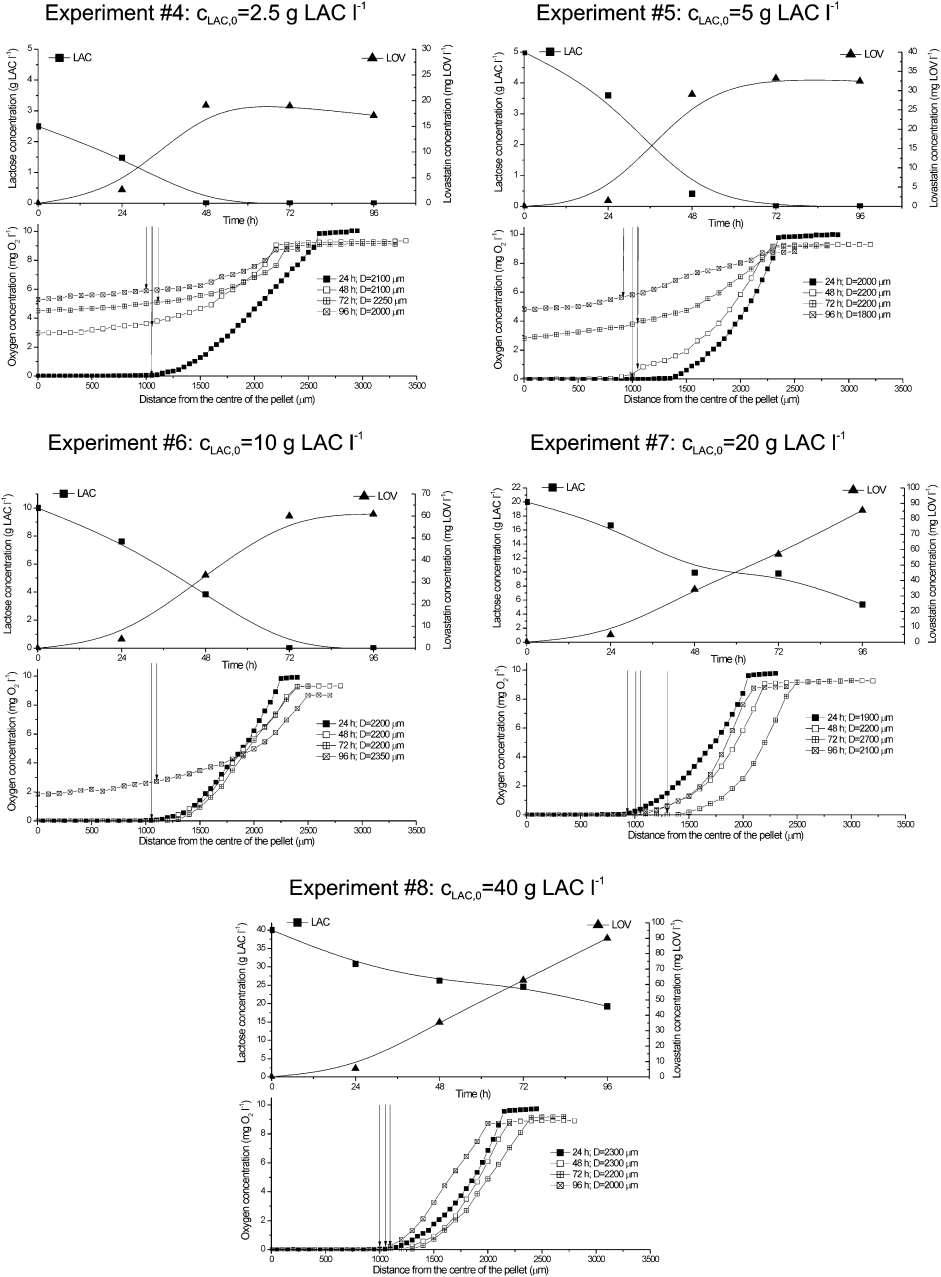
Time changes of averaged oxygen profiles in the viable pellets, lovastatin lactose concentration in experiments from #4 to #8; *vertical arrows* the surface of the pellet

Lactose concentration is of high importance for *A. terreus* and lovastatin, whose formation is strongly carbon substrate and C/N ratio dependent [12, 14]. It is also seen in Fig. 1a, c. Nevertheless, carbon substrate utilisation requires oxygen, namely electron acceptor, and the required amounts of these two important components are somewhat connected with each other. In the next series of experiments (from #4 to #8), the effect of varied initial lactose concentration was studied in association with oxygen transfer into pellets. In Fig. 6, oxygen profiles in the pellets and lactose and lovastatin concentrations are shown.

In experiments from #4 to #8, the pellets of mean diameter of 2,100 ± 200 μm were used. It was observed that at the lowest initial lactose concentrations c_LAC,0_ (up to 10 g l^−1^), intrapellet oxygen concentration had relatively high values, excluding the earliest hours of the experiment. Its reason was that the deficiency of lactose, actually there was no lactose in the broth after 48 h of experiment, made oxygen utilisation unnecessary. Oxygen diffused but it did not participate in the biological reaction. This fact was reflected in very low lovastatin titres (Fig. 6). The best results of lovastatin titres were obtained for the initial lactose concentrations of 20 and 40 g LAC l^−1^. In these runs, low or even zero oxygen concentrations inside the pellets were observed. Lovastatin titres were comparable, which proved that increasing of the initial lactose concentration had no sense because of oxygen deficiency. Also, at c_LAC,0_ = 40 g l^−1^, lactose was not ultimately utilised.

**Fig. 7:**
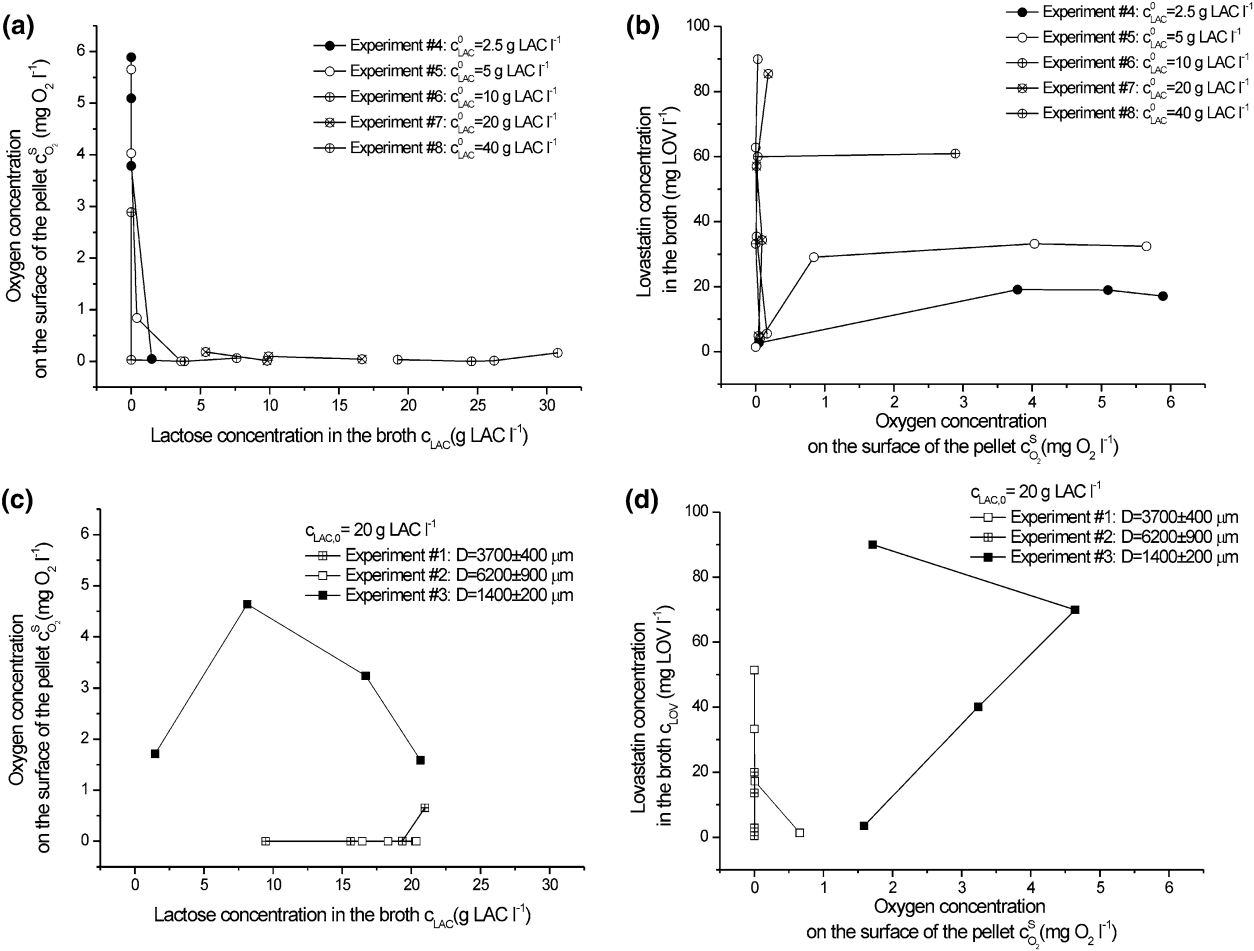
Correlation between oxygen concentration on the surface of the pellet and lactose concentration in the broth for experiments (**a**) from #4 to #8 and (**c**) from #1 to #3, and oxygen concentration on the surface of the pellet and lovastatin concentration in the broth for experiments (**b**) from #4 to #8 and (**d**) from #1 to #3

To more thoroughly study the influence of oxygen on lovastatin formation in association with lactose concentration, it was assumed upon the experiments from #1 to #3 and the consecutive quantitative analysis of oxygen transfer that the most important factor determining *A. terreus* growth and lovastatin formation was oxygen concentration on the surface of the pellets $c_{{{\text{O}}_{ 2} }}^{\text{S}}$. It influenced both biological reaction rate and was the outcome of the diffusive and convective oxygen transfer into the individual pellet. Thus, in Fig. 7, $c_{{{\text{O}}_{ 2} }}^{\text{S}}$ was related to lactose concentration in the broth and lovastatin titres for all experiments performed. At the high initial lactose concentrations (20 and 40 g l^−1^), $c_{{{\text{O}}_{ 2} }}^{\text{S}}$ was close to zero during the whole duration of the experiments #7 and #8 (Fig. 7a). But the decrease in pellet diameter by about 500 μm changed significantly this adverse trend, which is seen, when experiment #3 and #7 (Fig. 7a, c) were compared. Oxygen concentration on the surface of the pellet varied between 1.5 and 5 mg O_2_ l^−1^ in experiment #3 dependent on the time of the process. External mass transfer limitation was decreased to the high extent and intrapellet diffusion was much facilitated.

As mentioned earlier, even high oxygen concentrations on the surface of the pellet had no positive effect on lovastatin production, if they were caused by lactose deficiency (compare Fig. 7a, b). Only vertically lain experimental points in Fig. 7b indicated efficient lovastatin production (c_LOV_ = 85.45 mg LOV l^−1^ in experiment #7), although $c_{{{\text{O}}_{ 2} }}^{\text{S}}$ was then close to 0. In Fig. 7d, for the smallest studied pellets (experiment #3), both high oxygen concentrations on the surface of the pellet and even higher lovastatin titre in 96 h (90.03 mg LOV l^−1^) were observed.

All in all, upon the aforementioned results three important factors, strictly bound together, can be listed down for lovastatin biosynthesis by *A. terreus*. It is advised to hold them in their optimum values to supply its efficient production. These are: (1) pellet diameter, at least lower than 1,500 μm but the smaller, the better, (2) oxygen concentration on the surface of the pellet at higher than zero and (3) lactose concentration in the broth not higher than 20 g l^−1^ in the conditions of the batch experiments presented here.

Only some of these issues were previously addressed (only partially) in the literature.

With regard to pellet morphology, it was noticed that in some experiments performed here, the difference in pellets diameters was not very large. But only 500 μm (on average) difference in pellet diameter caused quite abrupt adverse effects with regard to oxygen transfer and lovastatin formation. Thus, pellet diameter occurred to be the most sensitive with regard to lovastatin biosynthesis process. What is more, technically it is not easy to obtain the pellets of the desired diameter with very high accuracy, even if spores were precisely enumerated before inoculation. The researchers working on lovastatin biosynthesis by *A. terreus* obtained the pellets of wide range of diameters. For the sake of clarity, the most significant literature data are collected in Table 2 together with the ones obtained in this work.

**Table 2:** *A. terreus* pellet diameters during lovastatin biosynthesis to be found in literature

Average pellet diameter (μm)	Lovastatin titre (mg LOV l^−1^)	Bioreactor*; carbon source; aeration/stirring	Literature
100–300	Not given	500-l STB; not given; controlled pO_2_ at 50 %	[5]
1,500 and 2,000	20 and 40	5-l STB; lactose; 800 and 300 rpm	[2]
2,000–3,000	Not given	17-l FBB; lactose; vvm = 1 l_iar_ l^−1^ min^−1^	[15]
750–1,600 650–1,350	331 458	125-ml ShF; lactose; shaking speed not given 5-l STB; lactose; vvm = 1 l_iar_ l^−1^ min^−1^, 225–425 min^−1^	[1]
3,500–5,650	900	2-l ALB; glucose and milk powder; 1.5 l_air_ l^−1^ min^−1^	[25]
1,000–3,800	From 30 to 70	150-ml ShF; lactose; 110 min^−1^	[6]
1,400–6,200	From 30 to 90	150-ml ShF; lactose; 110 min^−1^	This work

*STB* stirred tank bioreactor, *FBB* fluidised bed bioreactor, *ShF* shake flask, *ALB* air-lift bioreactor

From Table 2, it is impossible to find any correlation between pellet size and lovastatin titre, as various strains (mutants in [28]) and process conditions were used (e.g. oxygen-enriched air in [15]). What is more important, none of the cited authors referred quantitatively to the issue of oxygen transfer into pellets nor did they explicitly show the correlation between pellet size and lovastatin titre. But taking the wide range of reported pellet diameters into account, oxygen transfer must have had an effect on lovastatin titres. This issue was only discussed in general terms in some of them and sometimes optimum pellet diameter for lovastatin formation was suggested [1, 7, 27] or determined [6].

Although lovastatin formation is very sensitive to the amount of available lactose (carbon substrate), increasing of the initial lactose concentration in the batch cultures gave inadequate effects [12, 14] because of the insufficient amount of oxygen and it is the important finding of this work. Here, for the first time, surface oxygen concentration on the surface of the pellets together with carbon substrate concentration in the broth were indicated as the key factor influencing lovastatin biosynthesis by *A. terreus*. If the initial carbon substrate level had been chosen to be extremely high, some authors then intuitively used oxygen-enriched air. High lactose concentration (above 100 g l^−1^) used by Rodriguez Porcel et al. required aeration of the culture with the pellets of 2,000–3,000 μm with oxygen-enriched air (up to 80 %) to achieve high lovastatin titres [21]. Probably due to the same reasons, Lai et al. [10] suggested using oxygen vectors, namely aliphatic hydrocarbons as n-dodecane, in the so-called two-phase cultivation. Holding high oxygen saturation in the broth was also often connected with higher aeration rate and higher rotary speed of impeller. It negatively changed fungal morphology due to shear stress. This way to decrease pellet diameter and increase oxygen supply in the bioreactor did not assure better lovastatin yield [2].

On the other hand, Lai et al. did not claim that extremely high oxygen saturation in the broth was required for lovastatin production. In their experiments made with initial lactose concentration of 70 g l^−1^, they achieved better lovastatin titres at 20 % than at 40 % oxygen saturation [1]. Nevertheless, mean diameter of pellets used in their experiment (they measured the distribution of pellet size) was always below 2,000 μm. Adverse effects of too intensive culture aeration were also reported by Bizukojc and Ledakowicz [9] but in this work the size of pellets was not given.

It is easy to get high carbon substrate concentration in the medium but it is not easy to assure the adequate oxygen concentration to effectively utilise it. But there are several methods to facilitate oxygen transfer in *A. terreus* during lovastatin biosynthesis. All of them should be above all aimed at decreasing pellet diameter. Thereby, using a typical chemical engineering approach, either cultivation in the high turbulence conditions (increase of *k*_*S*_ *a* and $c_{{{\text{O}}_{ 2} }}^{\text{S}}$) or decrease of pellet diameter by shearing stress could be theoretically used but often without any guarantee of success as mentioned above [2]. Using oxygen-enriched air was a better idea [21]. But the alternative are morphological engineering techniques, the traditional and the new ones [29]. They occurred to be promising methods with regard to maximise lovastatin production by *A. terreus*. The classical manipulation with spore number in the preculture can be applied [30] and it occurred successful upon the data presented in this study and in the work of Bizukojc and Ledakowicz [6]. But in the case of lovastatin biosynthesis, another morphological engineering technique was more efficient. The addition of mineral microparticles (talc) changed the structure of the pellets, increasing biomass concentration, and decreased its diameter. It contributed to the increase of effective diffusivity and led to even twofold increase in lovastatin titre [11]. But in the actions described above, carbon substrate concentration in the broth must not be disregarded to avoid both overfeeding and starvation of *A. terreus* cells, when the structure and size of pellets were changed.

## Conclusions

The most important factor to efficiently produce lovastatin by *A. terreus* is to assure both the sufficient amount of carbon source and oxygen, especially when the mycelial growth in the pelleted form is observed. Overfeeding with carbon source (lactose) has no sense, if the insufficient amount of oxygen is supplied and oxygen concentration on the surface of the pellets tends to zero. On the other hand, the deficiency of carbon source ceases lovastatin production, even if there is enough oxygen. As the optimum oxygen concentration in the pellet, in association with carbon substrate concentration, strongly depends on pellet diameter, being the extremely sensitive parameter in lovastatin biosynthesis, it is important to fully control fungal morphology to obtain the optimum lovastatin titres.

## Appendix

From Eq. 3 Weisz’s modulus and from Eq. 4, internal efficiency for *A. terreus* pellets with assumed Michaelis–Menten biological kinetics was calculated. Thereby, the following values had to be earlier defined:

- ratio β
  8 $$\beta = \frac{{K_{\text{M}} }}{{c_{{{\text{O}}_{ 2} }}^{\text{S}} }}$$
  where: $c_{{{\text{O}}_{ 2} }}^{\text{S}}$ oxygen concentration on the surface of the pellet (mg O_2_ l^−1^) and K_M_ Michaelis constant (mg O_2_ l^−1^),
- generalised Thiele modulus *ϕ*_m_ for the sphere
  9 $$\phi_{\text{m}} = \frac{D}{6\sqrt 2 } \cdot \sqrt {\frac{{r_{\hbox{max} } }}{{D_{\text{eff}} \cdot c_{{{\text{O}}_{ 2} }}^{\text{S}} }}} \left( {\frac{1}{1 + \beta }} \right)\left[ {1 + \beta \ln \left( {\frac{\beta }{1 + \beta }} \right)} \right]^{ - 0.5}$$
  where: *D* pellet diameter (μm), *r*_max_ maximum rate of biological reaction (mg O_2_ l^−1^ s^−1^), *D*_eff_ effective diffusivity (μm^2^ s^−1^),
- internal efficiency for first-order kinetics *η*_i,1_
  10 $$\eta_{{{\text{i}},1}} = \frac{1}{{3 \cdot \phi_{1}^{2} }} \cdot \left( {3 \cdot \phi_{1} \cdot \coth \left( {3 \cdot \phi_{1} } \right) - 1} \right)$$
  where:
  $$\phi_{1} = \frac{D}{6} \cdot \sqrt {\frac{{k_{1} }}{{D_{\text{eff}} }}}$$
  is Thiele modulus for first-order reaction. As Michaelis–Menten kinetics was assumed, first-order rate constant *k*_1_ was calculated from:
  11 $$k_{ 1} = \frac{{r_{\hbox{max} } }}{{K_{\text{M}} }}$$
- internal efficiency for zero-order kinetics:
  12 $$\eta_{{\rm i},0} = \left\{ \begin{array}{ll} 1 & {\text{for}} \quad 0 < \phi_{0} \le 0.577\\ 1 - \left[ {\frac{1}{2} + \cos \left( {\frac{\varPsi + 4 \cdot \pi }{3}} \right)} \right]^{3} &{\text{for}} \quad \phi_{0} > 0.577 \\ \end{array} \right.$$
  where:
  13 $$\varPsi = \arccos \left( {\frac{2}{{3 \cdot \phi_{0}^{2} }} - 1} \right)$$
  and Thiele modulus for zero-order kinetics
  14 $$\phi_{0} = \frac{D}{6\sqrt 2 } \cdot \sqrt {\frac{{k_{0} }}{{D_{\text{eff}} \cdot c_{{{\text{O}}_{ 2} }}^{\text{s}} }}}$$
  Zero-order rate constant was calculated as:
  15 $$k_{0} = r_{\hbox{max} }$$

## List of symbols

*a*: Specific area of mass exchange (μm^2^ μm^−3^)
*A*_pellet_: Surface area of the pellet
*c*_LAC_: Lactose concentration (g LAC l^−1^)
*c*_LAC,0_: Initial lactose concentration (g LAC l^−1^)
*c*_LOV_: Lovastatin concentration (mg LOV l^−1^)
$c_{{{\text{O}}_{2}^{{}} }}$: Oxygen concentration (mg O_2_ l^−1^)
$c_{{{\text{O}}_{2} }}^{\text{B}}$: Oxygen concentration in the bulk liquid (mg O_2_ l^−1^)
$c_{{{\text{O}}_{2} }}^{\text{S}}$: Oxygen concentration on the surface of the pellet (mg O_2_ l^−1^)
*D*: Pellet diameter (μm)
*D*_eff_: Effective diffusivity (μm^2^ s^−1^)
*K*_M_: Michaelis constant (mg O_2_ l^−1^)
*k*_L_: Convective mass transfer coefficient between air bubble and liquid phase (s^−1^)
*k*_S_: Convective mass transfer coefficient between liquid phase and pellet (s^−1^)
*r*: Pellet radius (μm)
*r*_LAC_: Volumetric lactose uptake rate (g LAC l^−1^ h^−1^)
*r*_LOV_: Volumetric lovastatin formation rate (mg LOV l^−1^ h^−1^)
*r*_max_: Maximum reaction rate for oxygen (mg O_2_ l^−1^ s^−1^)
*r*_N_: Volumetric organic nitrogen uptake rate (g N l^−1^ h^−1^)
$r_{{{\text{O}}_{2} }}$: Biological reaction rate with regard to oxygen (mg O_2_ l^−1^ s^−1^)
*r*_X_: Volumetric biomass growth rate (g X l^−1^ h^−1^)
Sh: Sherwood number (–)
*V*_pellet_: Volume of the pellet (μm^3^)
*Φ*: Weisz’s modulus (–)
*β*: Ratio of Michaelis constant to oxygen concentration on the surface of the pellet (–)
*ϕ*_0_: Generalised Thiele modulus for zero-order biological kinetics (–)
*ϕ*_1_: Generalised Thiele modulus for first-order biological kinetics (–)
*ϕ*_m_: Generalised Thiele modulus for Michaelis–Menten biological kinetics (–)
*η*_i,0_: Internal efficiency for zero-order biological kinetics (–)
*η*_i,1_: Internal efficiency for first-order biological kinetics (–)
*η*_e,m_: External efficiency for Michaelis–Menten biological kinetics (–)
*η*_i,m_: Internal efficiency for Michaelis–Menten biological kinetics (–)
